# Screening of global microbiomes implies ecological boundaries impacting the distribution and dissemination of clinically relevant antimicrobial resistance genes

**DOI:** 10.1038/s42003-022-04187-x

**Published:** 2022-11-18

**Authors:** Qiang Lin, Basil Britto Xavier, Blaise T. F. Alako, Alex L. Mitchell, Sahaya Glingston Rajakani, Youri Glupczynski, Robert D. Finn, Guy Cochrane, Surbhi Malhotra-Kumar

**Affiliations:** 1 Laboratory of Medical Microbiology, Vaccine & Infectious Disease Institute, University of Antwerp, Antwerp, Belgium; 2 European Molecular Biology Laboratory, European Bioinformatics Institute, Wellcome Genome Campus, Cambridge, UK

**Keywords:** Antimicrobial resistance, Microbiology

## Abstract

Understanding the myriad pathways by which antimicrobial-resistance genes (ARGs) spread across biomes is necessary to counteract the global menace of antimicrobial resistance. We screened 17939 assembled metagenomic samples covering 21 biomes, differing in sequencing quality and depth, unevenly across 46 countries, 6 continents, and 14 years (2005-2019) for clinically crucial ARGs, mobile colistin resistance (*mcr*), carbapenem resistance (CR), and (extended-spectrum) beta-lactamase (ESBL and BL) genes. These ARGs were most frequent in human gut, oral and skin biomes, followed by anthropogenic (wastewater, bioreactor, compost, food), and natural biomes (freshwater, marine, sediment). *Mcr-9* was the most prevalent *mcr* gene, spatially and temporally; *bla*_*OXA-233*_ and *bla*_*TEM-1*_ were the most prevalent CR and BL/ESBL genes, but *bla*_*GES-2*_ and *bla*_*TEM-116*_ showed the widest distribution. Redundancy analysis and Bayesian analysis showed ARG distribution was non-random and best-explained by potential host genera and biomes, followed by collection year, anthropogenic factors and collection countries. Preferential ARG occurrence, and potential transmission, between characteristically similar biomes indicate strong ecological boundaries. Our results provide a high-resolution global map of ARG distribution and importantly, identify checkpoint biomes wherein interventions aimed at disrupting ARGs dissemination are likely to be most effective in reducing dissemination and in the long term, the ARG global burden.

## Abstract (web summary)

An extensive metagenomic analysis investigates the global distribution of antimicrobial resistance genes over different biomes, which are primarily influenced by host genera and environment rather than geographic or anthropogenic factors.

## Introduction

Antibiotic resistance claims high morbidity, mortality and economic costs^1–3^, and is a global health threat. This threat is exacerbated by the global dissemination of human and animal pathogens harboring mobile antibiotic-resistance genes (ARGs)^4^, in particular those conferring resistance to colistin, and beta-lactams including carbapenems that are considered as antibiotics of last resort for human infections due to multidrug-resistant bacteria^5,6^. The transfer of these ARGs across microorganisms broadens their range of host species, thus allowing a wide existence of these genes in various biomes/habitats. Prior studies on ARGs have mainly focused on specific biomes (e.g., human gut^7^, sewage^8^, farms^9^, estuary sediments^10^, and soil^11^), but a global One Health (Human–Animal–Environment) ARG landscape across diverse biomes (human, animal, plant, anthropogenic environment and natural environment) is lacking. Comparing ARGs profiles across diverse biomes provides an unique opportunity to comprehensively understand global prevalence and the preferred niches of ARGs, and is a prerequisite for the development of effective and targeted countermeasures against ARG spread.

The enormous amount of antibiotics that are regularly released into the environment could generate selection to govern the dissemination and fate of ARGs across biomes; a recent survey reported that 53,800 tons of antibiotics were released into the environment in China alone during 2013^12^. Physical and biological forces (e.g., wind, river, and animal migration)^13^ and human mobility^14^ could further weaken the geographic barriers for ARG dissemination. The characteristics of biomes where ARGs exist could have an environmental selection on ARGs-harboring microorganisms and prophages^15,16^; the phylogeny of such microorganisms could influence ARGs distribution^17^; genetic elements (plasmid, insertion sequences, and integrons) could promote horizontal gene transfer of ARGs across microorganisms^15^; or ARGs distribution could be random due to stochastic processes (e.g., random birth/death events, uncertain dispersal for random chance of microbial colonization, ecological drift, and environmental disturbance^18^). Thus, although various forces could drive the distribution and dissemination of ARGs, their relative importance in ARG distribution and dissemination in a global context is barely explored.

During the last decades, worldwide efforts have generated tremendous amounts of metagenomic data from various biomes across geographic and temporal distributions, and deposited in public repositories (e.g., European Nucleotide Archive (ENA)). These metagenomic datasets provide us with an unprecedented opportunity to perform a One Health global surveillance of some of the most important mobile ARGs with respect to human and animal health: mobile colistin resistance genes (*mcr*, genes encoding enzymes conferring resistance to colistin that is an antibiotic of last resort to treat multidrug-resistant Gram-negative infections), carbapenem resistance genes (CR, genes encoding enzymes conferring resistance to carbapenems that are used to treat multidrug-resistant infections), and (extended-spectrum) beta-lactamase genes (ESBL and BL, genes encoding enzymes conferring resistance to beta-lactam antibiotics, one of the largest and most commonly used class of antibiotics). Here, all shotgun metagenomic datasets deposited at ENA and other public databases until October 30, 2019 (*n* = 17,939 samples) were screened for prevalence and distribution of all known mobile ARGs encoding resistance to last-line antibiotics: colistin, and beta-lactams including carbapenems. The metagenomic datasets included samples spanning 21 biomes, 46 countries, 6 continents, and 14 years from 2005 to 2019.

We hypothesized three scenarios: (1) these ARGs distribute and disseminate globally, because physical and biological forces and human mobility could weaken geographic barriers and horizontal gene transfer could weaken genetic barriers, which together promote the global spread of ARGs; (2) the distribution and dissemination of ARGs are shaped by deterministic processes (i.e., ecological boundaries), because host species of these genes are framed by ecological processes (e.g., biotic and abiotic selection and interactions that determine the distribution and dissemination of ARGs-harboring microorganisms)^15,16^; (3) the distribution and dissemination of ARGs is random due to stochastic processes (e.g., random microbial birth/death events, random chance of microbial colonization, ecological drift and environmental disturbance^18^).

We find that the global distribution of the targeted ARGs follows a non-random pattern and is primarily shaped by the biomes where they exist and their potential host species, with transmission preferentially occurring between biomes with similar characteristics within defined ecological boundaries.

## Results

### Global distribution of resistance genes

**Fig. 1:**
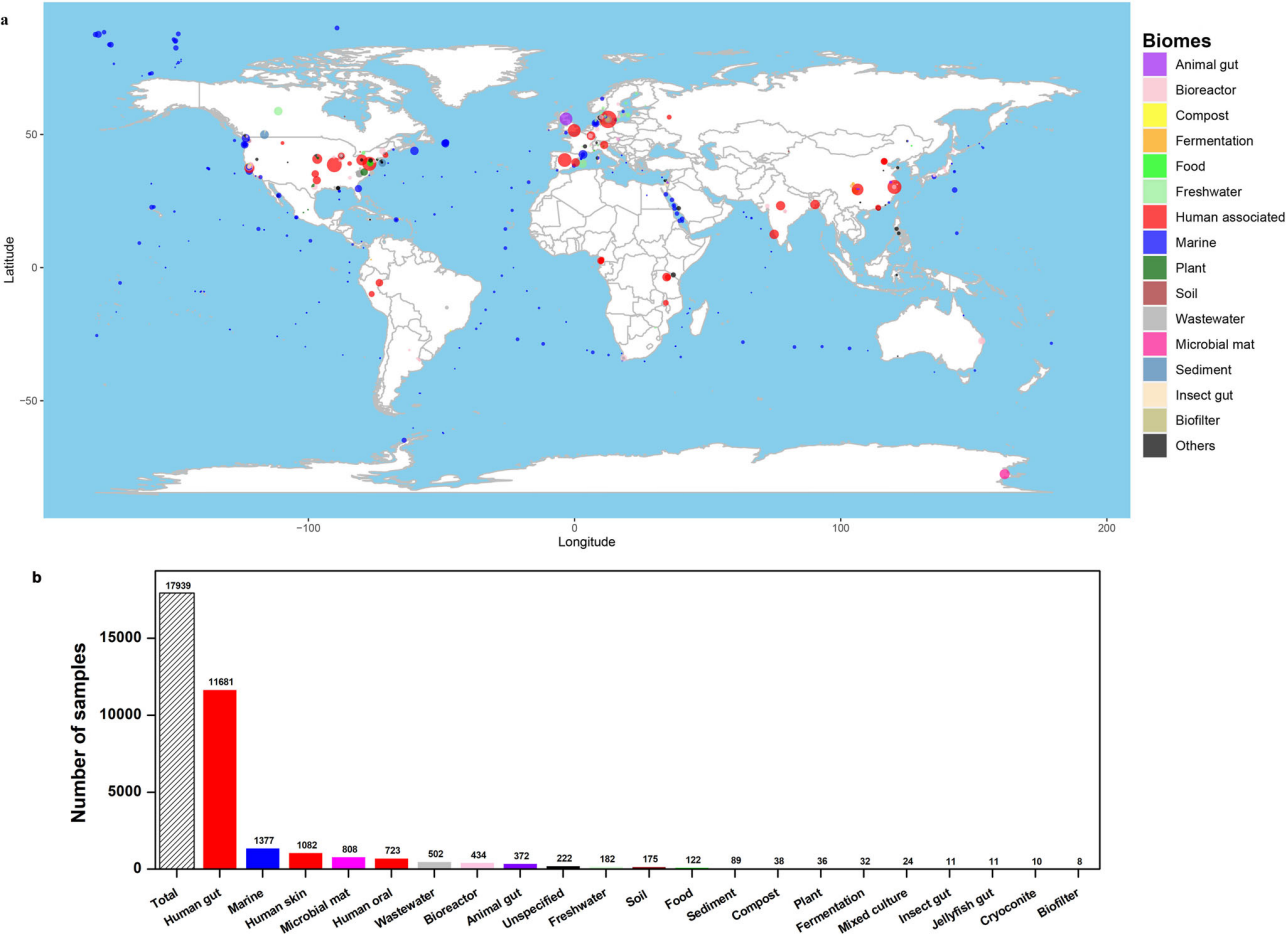
Globally screened samples. **a** Global distribution of screened samples. Nodes are colored by different biomes, and sized by the number of samples. Human-associated biomes include human gut, human skin and human oral. Only samples with available location information are shown. **b** Biome distribution of screened samples.

The *mcr*, CR, and ESBL/BL (ESBL or BL) genes were queried against the 17939 metagenomic assembled samples which were collected from 2005 − 2019, and across 6 continents covering 46 countries (e.g., USA (*n* = 2925), China (*n* = 1802), Denmark (*n* = 714), UK (*n* = 500) and Spain (*n* = 445)) (Fig. 1a). These samples cover 21 typical biomes, including human-associated biomes (*n* = 13,486) (gut, skin, and oral), anthropogenic biomes (*n* = 1136) (wastewater, bioreactor, food, compost, fermentation, and biofilter), natural biomes (*n* = 1641) (marine, soil, freshwater, and sediment), animal gut (*n* = 372), plant (*n* = 36), and others (*n* = 1268) (Fig. 1b). Among the metagenomic samples, 746 (4.16%) samples were found to harbor BL/ESBL genes, 85 (0.47%) samples were detected to harbor CR genes, and 51 (0.28%) samples harbored *mcr* genes.

**Fig. 2:**
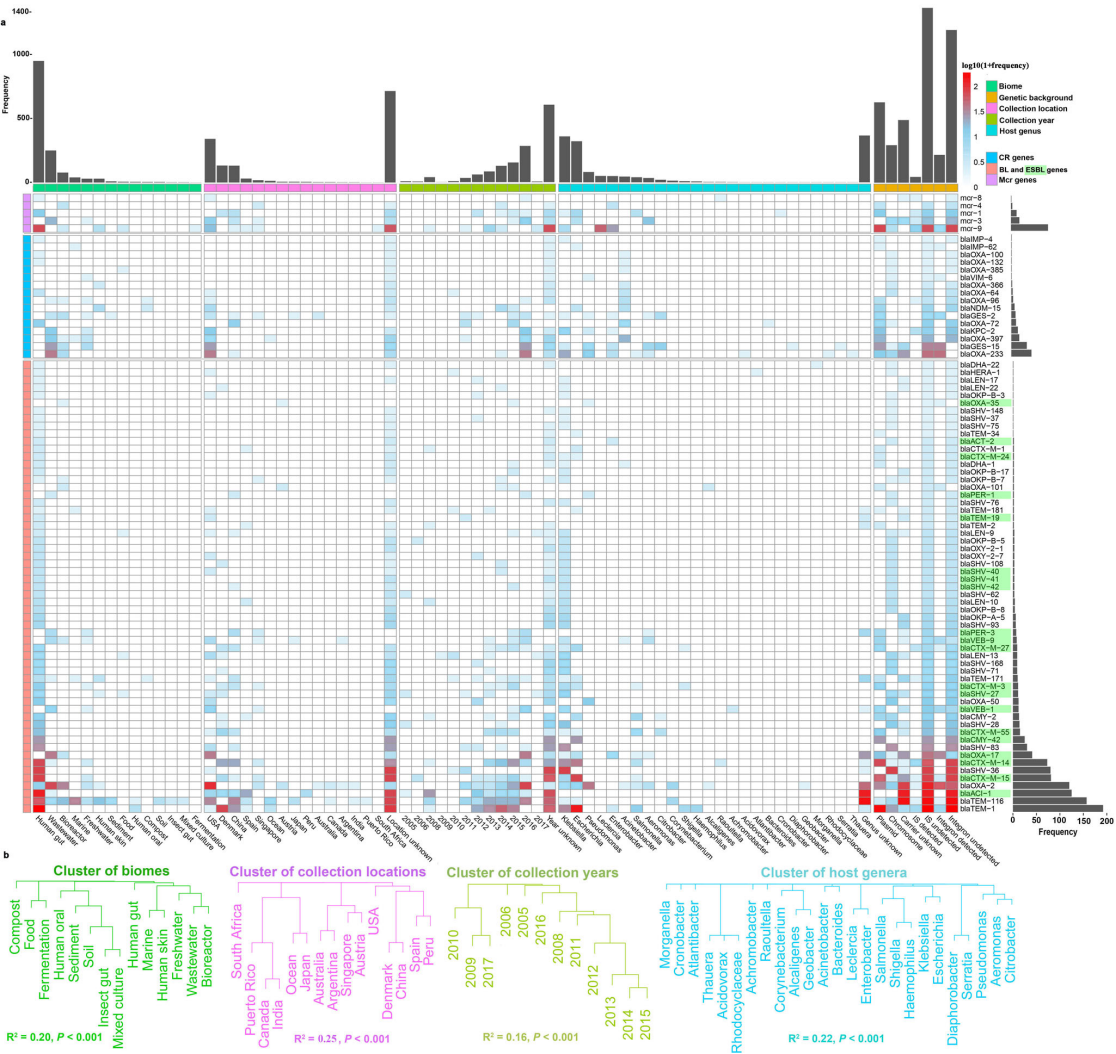
Distribution of individual resistance genes. **a** Heatmap shows frequency (number of hits) of individual resistance genes across biomes, collection locations, collection years, host genera and genetic backgrounds. Cells are colored by log_10_(1+frequency). The bar plot above shows the total frequency of *mcr*, CR, BL and ESBL genes across biomes, collection locations, collection years, host genera and genetic backgrounds, and the bar plot on the right shows the frequency of individual resistance genes. ESBL genes are marked in green. **b** Dendrograms show the clusters of these detected genes across biomes, collection locations, collection years and host genera, respectively. The values of R^2^ and *P* evaluated in the permutational multivariate analysis of variance with 999 permutations are shown below the dendrograms.

Only 5 *mcr* gene variants were detected in samples collected during 2008-2017 from 7 biomes and 6 countries (Fig. 2a and Supplementary Fig. 1). Of the *mcr*-harboring scaffolds (assembled sequences from metagenomic short reads harboring *mcr* genes), 80% were plasmid-mediated, 44% and 24% were potentially hosted by the genus *Leclercia* and *Enterobacter*, and 16% and 8% carried insertion sequences (IS) and integrons, respectively (Fig. 2a). *Mcr-9* was most frequently detected (79 hits (sequences mapped)) and most prevalent spatially (across 5 countries, mainly in USA), temporally (spanning 5 years, dating back to 2008 in a human gut sample collected in Spain) and across diverse biomes (mainly in human gut) (Fig. 2a). *Mcr*-3 variants (18 hits mainly in wastewater), *mcr-1* (12 hits in human gut), *mcr*-*4* (3 hits in bioreactor) and *mcr-8* (1 hit in human gut) were also detected.

Seventeen CR gene variants were detected in samples collected during 2010 − 2016 from 9 biomes and 5 countries (Fig. 2a and Supplementary Fig. 2). Of the CR-harboring scaffolds, 68% were plasmid-mediated, 28% and 24 % were potentially hosted by the genus *Acinetobacter* and *Klebsiella*, and 3% and 59% carried IS and integrons (Fig. 2a). *Bla*_*OXA-233*_ (43 hits), *bla*_*GES*-*15*_ (33 hits), *blaOXA*-*397* (17 hits) and *bla*_*KPC*-*2*_ (14 hits) were frequently detected but were restricted mainly to wastewater samples collected from USA in 2016 (Fig. 2a). *Bla*_*GES*-*2*_ gene (9 hits) was most prevalent spatially (across 5 countries) and temporally (spanning 4 years), and present in 4 biomes (e.g., wastewater and bioreactor) (Fig. 2a).

Fifty-nine BL/ESBL gene variants were detected in samples collected during 2005 − 2016 from 14 biomes and 13 countries (Fig. 2a and Supplementary Fig. 3). Of the BL/ESBL-harboring scaffolds, 38% were plasmid-mediated, 28% and 27% were potentially hosted by the genus *Klebsiella* and *Escherichia*, and 2% and 11% carried IS and integrons (Fig. 2a). *Bla*_*TEM-1*_ (192 hits) was most frequently found in human gut, followed by *bla*_*TEM-116*_ (157 hits) that was most prevalent spatially (across 8 countries, mainly in USA and China, Supplementary Fig. 4), temporally (spanning from 2008 to 2016) and across 11 biomes (mainly in human gut and marine) (Fig. 2a).

The ARGs profiles differed significantly across different biomes, collection locations, collection years and potential hosts, respectively (*p* < 0.001 for each, Fig. 2b and Supplementary Fig. 5-7). Gene frequency (number of gene hits) significantly and positively correlated with the number of biomes, collection locations, collection years and potential hosts, respectively (*p* < 0.01 for all, Supplementary Fig. 8).

Among anthropogenic factors, only gross domestic product (GDP) and domestic education level significantly affected the global distribution of these ARGs (*p* < 0.05, Supplementary Data 1). Further, null model results showed that the separate distributions of *mcr*, CR, and BL/ESBL genes differed significantly (FDR-adjusted *p* < 0.001 for each) from the corresponding random distributions, indicating that the observed distributions of these ARGs were deterministic (i.e., non-random).

**Fig. 3:**
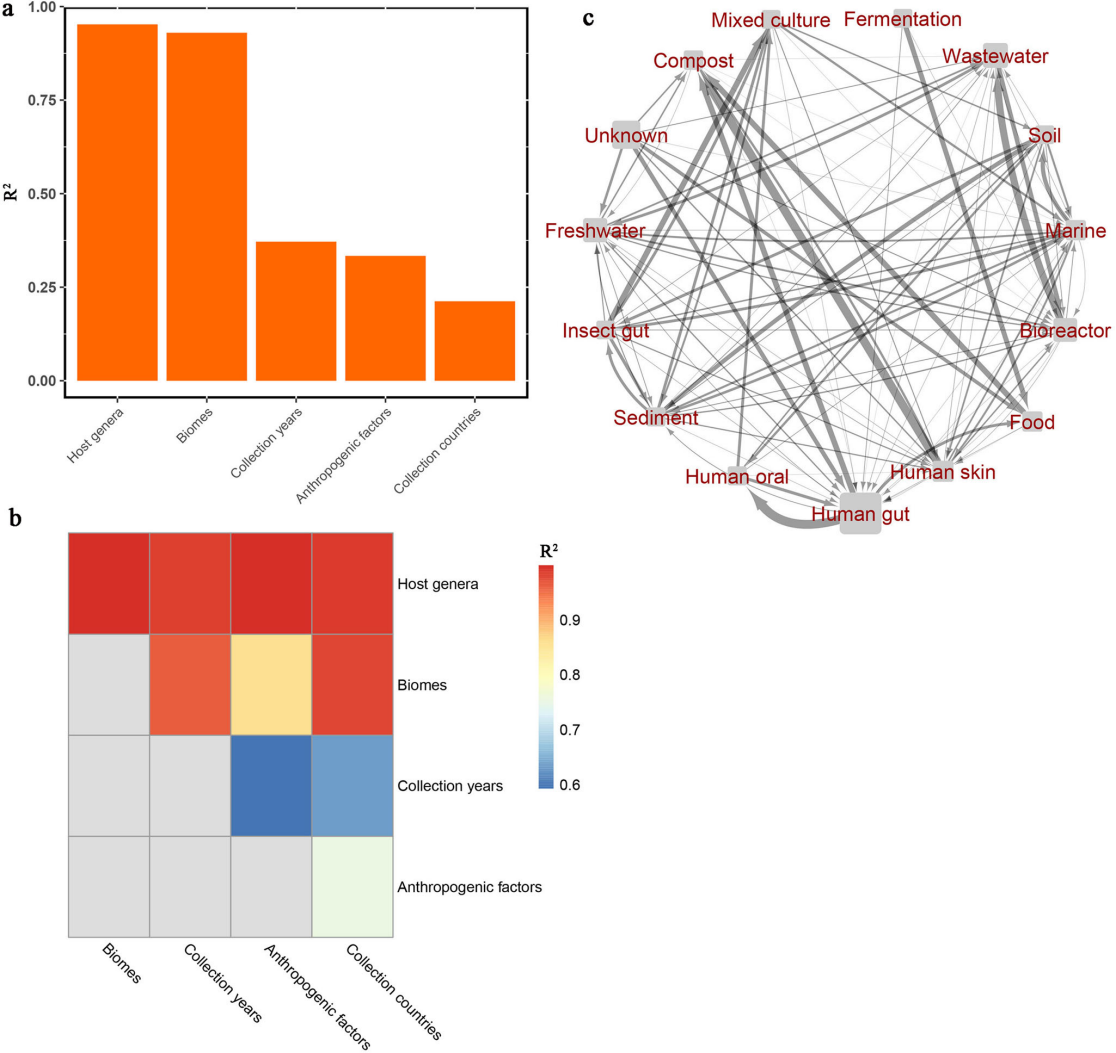
Effects of host genera, biomes, collection year, anthropogenic factors (GDP, urban ratio, total population, education level, agricultural land use, total greenhouse gas emissions and antibiotics use) and collection countries on the distribution of all the detected ARGs. **a** Separate effects of variables. **b** Conjoint effects of two variables. Separate and conjoint effects are evaluated by the R^2^ values in the redundancy analysis. **c** The Bayesian inference network (c) portrays the dissemination of all the detected ARGs across biomes. Based on a hypothesis that any biomes can be the source biomes that transmit ARGs to other biomes, as well as the sink biomes that accept ARGs from other biomes directly or indirectly, an arrow is used to represent the dissemination path from the source biome to the sink biome. Line width is based on the estimated transmission proportion of ARGs from the source biome to the target biome, and the rectangle representing the biome is sized by the number of ARGs variants harbored in the biome. “Unknown” means no specific source biomes.

Redundancy analysis (RDA) showed that the global distribution of the ARGs was best explained by potential host genera (95%) and biomes (93%), followed by collection year (37%), anthropogenic factors (33%) and collection countries (21%) (Fig. 3a). The conjoint effect of potential host genera and biomes was the highest amongst all other conjoint variables (Fig. 3b).

### Prevalence and potential dissemination of resistance genes across biomes

**Fig. 4:**
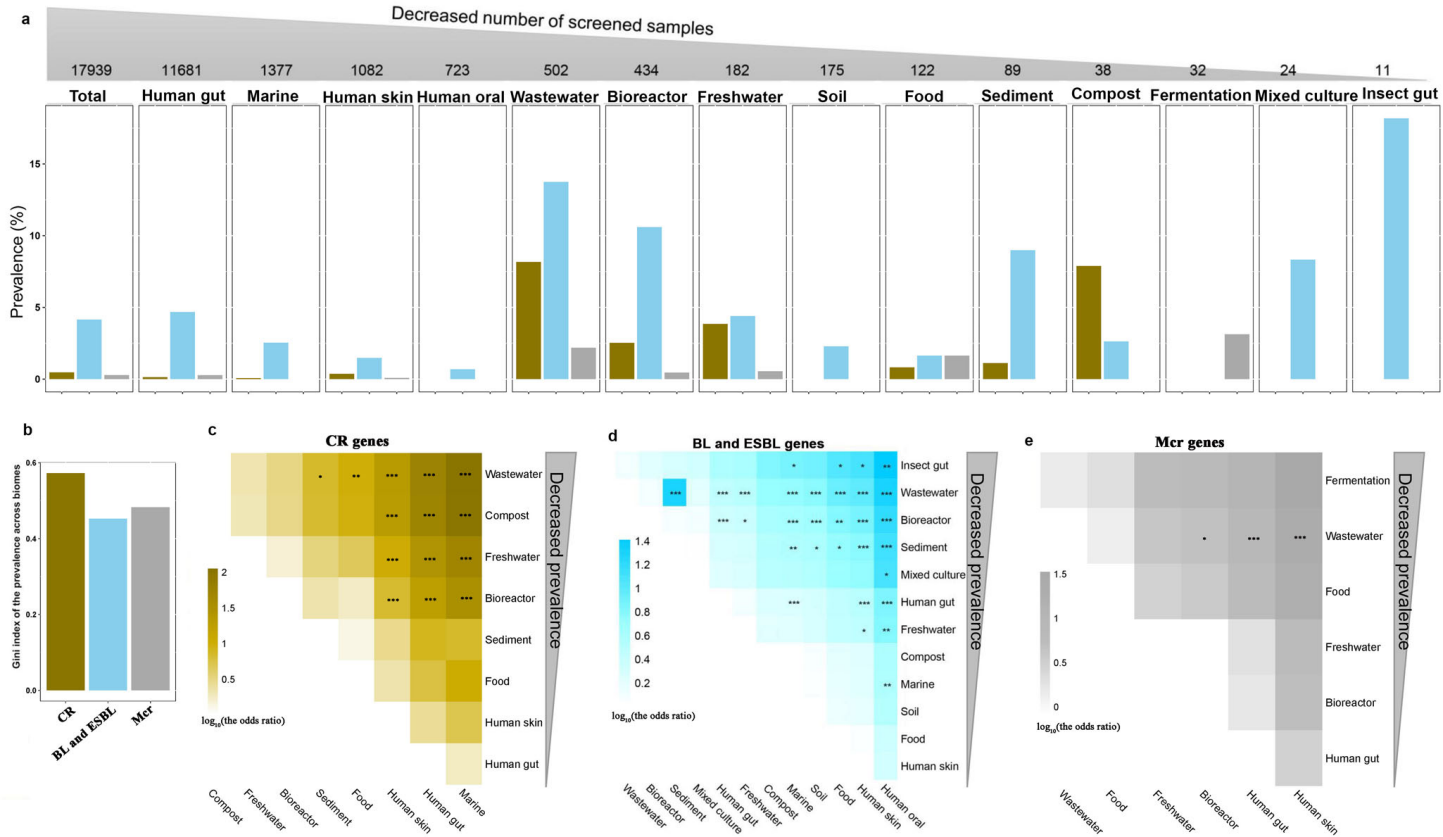
Gene prevalence across biomes. **a** Gene prevalence in a specific biome is determined by dividing the number of samples (in the biome) where the ARGs are detected by the number of total screened samples in the biome. **b** Gini index evaluates the inequality of the ARGs prevalence distribution across biomes. **c** The differences in prevalence between biomes for CR genes. **d** The differences in prevalence between biomes for BL and ESBL genes. **e** The differences in prevalence between biomes for *mcr* genes, evaluated by the Fisher’s Exact Test. The cells in heatmaps are colored by log_10_(the odds ratio), and *, **, and *** represent differences of prevalence between two biomes at significance with *p* < 0.05, *p* < 0.01, and *p* < 0.001, respectively.

In most biomes, BL/ESBL genes showed a higher prevalence than *mcr* and CR genes (Fig. 4a). A lower Gini index for BL/ESBL prevalence than *mcr* and CR prevalence indicated BL/ESBL genes were more evenly prevalent across biomes than *mcr* and CR genes (Fig. 4b). Amongst biomes, wastewater showed significantly higher prevalence of BL/ESBL (13.75%), *mcr* (2.19%) and CR (8.17%) genes than other biomes (*p* < 0.05 for all, Fisher’s exact test, Fig. 4c–e). CR (7.89%) and BL/ESBL (10.60%) genes were also highly prevalent in compost and bioreactor biomes, respectively (Fig. 4c, d).

Bayesian analysis based on Bayesian inference showed that nearly all the detected biomes could be the potential source biomes as well as the sink biomes for ARG transmission, but with very different transmission proportions between potential source-sink biome pairs (Fig. 3c). ARGs from up to 14 source biomes could be potentially transmitted to human gut and wastewater. Human gut was identified as the major source biome (80%) of ARGs when the sink biome was the human oral biome. Bioreactor and wastewater were the major source (40%) and sink (60%) biomes for ARG transmission between each other.

Upon classifying biomes into natural, anthropogenic and human-associated biomes, the transmission proportions of ARGs between natural and anthropogenic biomes (45% each and in both directions, respectively) and between natural and human-associated biomes (16% and 43% in both directions, respectively) were higher than that between anthropogenic and human-associated biomes (6% and 10% in both directions, respectively) (Supplementary Fig. 9). These results indicate that natural biomes might act as a potential transmission bridge of ARGs between anthropogenic and human-associated biomes.

## Discussion

This study presents a comprehensive screening of publicly available and global metagenomic datasets (until October 2019) to compile a landscape of the distribution of *mcr*, CR, and BL/ESBL genes. We study here diverse biomes in a global context representing diverse proximities to hotspots of antibiotic use and identify a decreasing frequency of these ARGs from human-associated biomes, to anthropogenic and natural biomes with a deterministic distribution pattern. Our findings imply ecological boundaries on the global distribution of ARGs across biomes.

### Ecological boundaries determine the distribution of *mcr* and CR genes

Although a widespread and even distribution of ARGs across biomes is expected because geographic barriers could be weakened by physical and biological forces and human mobility^13^, we detected a decreasing frequency of ARGs from human-associated biomes (988 hits) to anthropogenic (342 hits) and natural biomes (55 hits). Besides these factors, ecological boundaries (different biomes have different abiotic and biotic characteristics for niche filtering of microorganisms^19^) might also determine the immigration of ARGs-harboring microorganisms across biomes, thus influencing global distribution and dissemination of ARGs. Additionally, the fitness costs induced by resistance gene acquisition (e.g., through horizontal gene transfer) and out-competition by antibiotic-susceptible bacteria^20^ might boost potential exclusion of the migrant microorganisms in the new environment, thus amplifying the effects of ecological boundaries. The importance of ecological boundaries in ARG distribution was supported by our finding that the individual effects of biomes and potential hosts, and their conjoint effects showed the most important influence on ARG distribution. The effects of potential hosts are also related to ecological boundaries, as the distribution of host microorganisms is largely determined by their niches. The effect of microbial phylogeny on resistomes has been reported in prior studies^9,17,21^, and our results of potential hosts further extend the prior finding from specific biomes to more diverse biomes. Null model results suggested that the distribution of ARGs was not driven by stochastic processes but by deterministic processes (abiotic and biotic selection^22^), supporting the importance of ecological boundaries. Unexpectedly, anthropogenic factors showed relatively limited effects on ARG distribution. This could possibly be biome-scale dependent. A single-biome study in urban sewage found a crucial role of anthropogenic factors in ARG global distribution^8^. Here, we integrate 21 globally-distributed biomes and highlight the key role of ecological boundaries. Additionally, anthropogenic factors are macroscopic indices at country-level, and are not directly linked to local biotic and abiotic characteristics to affect resistomes. Anthropogenic factors were found to play an important role in pharmaceutical pollution of rivers in lower-middle income countries^23^, while our study had relatively low numbers of samples from such countries. It is thus conceivable that the uneven distribution of our global datasets across countries possibly hinders the revelation of the link between anthropogenic factors and ARG distribution. Collectively, these could explain the relatively limited effects of anthropogenic factors on ARG distribution observed in this study.

An uneven distribution of ARG prevalence across different biomes was observed. ARGs were most prevalent in wastewater and thus wastewater might be a hotspot for ARGs exchange, in line with prior reports^24^. Therefore, wastewater is a checkpoint biome for interventions aimed at disrupting ARGs dissemination. However, a prior study found that the freshwater biome is a hotspot for gene exchange^25^. The disagreement is probably because our study focused on ARGs, while the prior study analyzed reads of a more extended spectrum of genes^25^. Wastewater represents a confluence of antibiotics, disinfectants, metals and nutrients from different environments (households, hospitals, factories, and others)^24^, which expands the diversity of microorganisms and ARGs here. Additionally, co-selection from antibiotics, disinfectants and metals, and the relatively lower concentrations of antibiotics in wastewater than in clinical settings could promote the chance of ARG existence and persistence in wastewater^26,27^.

Our finding of a low prevalence and limited distribution of *mcr* genes is not in line with a previous report of a “substantial unseen resistome”^28^, and is inconsistent with the inference of widespread *mcr* genes by a retrospective report^29^. Samplings performed in proximity to environments where antibiotics are frequently consumed like hospitals, farms etc. increase the probability of identifying ARGs^13^. This might be one of the reasons explaining the inconsistency between our findings and prior reports, as the global datasets used in this study consist of samples from diverse biomes with diverse proximities to human activities and hotspots of antibiotic use. Nonetheless, the proposed “substantial unseen resistome”^28^ complements our data in putting forward a hypothesis of ecological boundaries. From a higher *mcr* prevalence setting where colistin is frequently implemented, the spread of *mcr*-harboring microorganisms to surrounding environments where colistin is less implemented will be subject to environmental dilution and filtering, which probably leads to a decreasing gradient of *mcr* gene prevalence as observed in our study.

Surprisingly, *mcr-9* showed the widest distribution across biomes and timespan (dating back to 2008 in a human gut sample collected in Spain), compared to its 8 earlier discovered homologs (*mcr-1* to *mcr-8*). This recently (2019) discovered *mcr-9* gene is an inducible gene under colistin exposure^30^, which suggests that *mcr-9* gene would remain unexpressed or be barely expressed in colistin free or low-concentration environments, consequently reducing waste of cellular energy and thus, fitness costs to facilitate wide distribution of *mcr-9* harboring microorganisms. This inference is supported by the fact that biomes (e.g., food and fermentation) where *mcr-9* was detected are unlikely to have been subjected to a direct colistin selection pressure.

### Potential transmission of ARGs across biomes

Although ARGs could potentially be transmitted among different biomes, the transmission proportions were higher between biomes with similar characteristics, such as between human gut and oral, and between wastewater and bioreactor, again underscoring the importance of ecological boundaries. Between biomes with dissimilar characteristics, the transmission proportions were very low, while a prior study finds a high degree of cross-biome mobility of genes^25^. The disagreement could possibly be attributed to different genes targeted between the two studies. Our findings provide evidence that natural biomes, rather than anthropogenic biomes, represent the major sink biomes as well as the major potential source biomes for human-acquired ARGs.

*Mcr-9* was found in food, human gut, human skin, fermentation and bioreactor, which implied the potential transmission routes of this ARG across these biomes. The transmission potential of *mcr-9* is probably attributed to the genetic background of *mcr-9* harboring scaffolds. Ninety-five percent of these scaffolds were mediated by plasmids, and some of them were flanked by IS elements, indicating potential for mobility of these *mcr-9* harboring scaffolds. Additionally, 63% of *mcr-9* harboring scaffolds shared similar genetic background with *Leclercia* species in our study, suggesting that *Leclercia* species were the potential hosts of *mcr-9* across biomes. The wide niche of *Leclercia* species^31,32^ further supports the transmission potential *mcr-9* across diverse biomes. Besides *mcr-9*, only *mcr-3* among *mcr* genes was found in wastewater and freshwater. *Mcr-3* has not been reported previously in these biomes, although it is found frequently in human and animal gut samples^33,34^. *Mcr-3* harboring scaffolds were potentially hosted by *Aeromonas* species that frequently exist in wastewater and freshwater^35,36^, supporting the transmission potential of *mcr-3* between these two environments. Remarkably, *bla*_*TEM-116*_ encoding beta-lactamases showed the widest distribution compared to the other queried genes, in line with prior reports of the widespread dissemination of this gene^37^. Most of the *bla*_*TEM-116*_ harboring scaffolds detected in the human gut in this study were potentially hosted by *Corynebacterium* species, some of which are known opportunistic human pathogens. Importantly, *bla*_*TEM-116*_ (ARO: 3000979) is a broad-spectrum beta-lactamase, harbored by multiple plasmids and isolated from numerous human pathogens. It is also the progenitor of more than 50 successful variants, establishing its centrality in the TEM family of beta-lactamases^38^. The potential transmission of *bla*_*OXA-233*_, *bla*_*GES-2*_, *bla*_*GES-15*_ and *bla*_*KPC-2*_ across biomes was also attributed to the genetic backgrounds, as most scaffolds harboring these genes were mediated by plasmids and IS elements, and were potentially hosted by microorganisms with wide niches’ breadth.

Our global-scale surveillance of ARGs had to take into account certain limitations in unevenness in sequencing quality, depth, and sample sizes across years, countries and biomes, which remains intractable for studies based on global datasets. In order to minimize the impact of these limitations on our results, we used a very strict threshold to confirm the presence of ARGs and applied normalized gene prevalence. Further, the non-significant correlation between scaffold length and sample collection year (Supplementary Fig. 10) implied only a minor impact of sequencing quality unevenness on our results. Our evaluation of the effects of anthropogenic factors on ARG distribution could not include important factors such as sanitation and drinking water supply due to lack of availability of complete datasets and warrant consideration in future studies. Despite these limitations, this study presents a high-resolution global map of the most important ARGs encoding resistance to last-line antibiotics. We show here that the global distribution of the targeted ARGs follows a non-random pattern and is primarily shaped by the biomes where they exist and their potential host species, with transmission preferentially occurring between biomes with similar characteristics defined by ecological boundaries. This study elucidates the mechanisms underlying the global distribution of ARGs of clinical importance, and the application of these mechanisms in context of other ARGs warrants further studies. Importantly, we identify checkpoint biomes wherein interventions aimed at disrupting clinically relevant mobile ARGs, such as those studied here, are likely to be most effective in reducing dissemination and in the long term, their global burden.

## Methods

### Data collection and process

To perform global surveillance of all known mobile colistin resistance-encoding (*mcr*) genes, carbapenem resistance-encoding genes (CR) and genes encoding beta-lactamase (BL) or extended spectrum beta-lactamase (ESBL) (Supplementary Data 2), we screened all publicly available assembled shotgun metagenomic data deposited in European Nucleotide Archive (ENA)^39^ and other public databases (Supplementary Data 3) (until October 30, 2019, *n* = 17,939 samples), covering 21 typical biomes: human-associated (*n* = 13,486), marine (*n* = 1377), wastewater (*n* = 502), bioreactor (*n* = 434), animal gut (*n* = 372), food (*n* = 122), freshwater (*n* = 182), soil (*n* = 175), and others (*n* = 1289), and distributing in 46 countries (e.g., USA (*n* = 2925), China (*n* = 1802), Denmark (*n* = 714), UK (*n* = 500), and Spain (*n* = 445)) across 6 continents, and spanning 14 years from 2005 to 2019. All public databases utilized in this study are listed in Supplementary Data 3. All study accession IDs, and the sample IDs where we identified the queried ARGs, are listed in Supplementary Data 4.

The full-length protein sequences of *mcr*, CR, BL and ESBL genes (Supplementary Data 2) were queried against the scaffolds (length ≥750 bp) in assembled metagenomic data by tblastn (searching translated nucleoside database using a protein query) with *E*-value (describing the number of hits one can expect to see by chance when searching a database of a particular size) ≤1 × 10^−5^. To confirm the presence of queried genes and to avoid the possibility of biased results caused by differences in sequencing depth and in the assembly tools across the collected assembled metagenomic samples, gene hits instead of gene abundances were used here. Hits of queried genes were only achieved by requesting 100% coverage (the percentage of a queried sequence aligned to the hit sequence) and ≥98% similarity of protein sequences of queried genes. In this context, the hits were determined despite the lengths of scaffolds or acquired genes, so the number of hits were not normalized by lengths of scaffolds or acquired genes. The hit-carrying scaffolds (Supplementary Data 4) and associated metadata were extracted (e.g., geographic and temporal information, and biomes) for further analyses. For these hit-carrying scaffolds, potential host species (that share similar genetic background with the scaffolds), plasmid- or chromosome-mediation, insertion sequences (IS) and integrons were explored by blastn against NCBI’s nr database^40^, ISfinder database^41^ and the INTEGRALL database^42^. The best hit of each scaffold was determined with *E*-value <10^−6^ and queried coverage ≥90% and similarity ≥90%. Data on anthropogenic factors (gross domestic product (GDP), urban ratio, total population, education level, agricultural land use, total greenhouse gas emissions, and antibiotics use) were collected from the World Bank (https://databank.worldbank.org/home.aspx), ResistanceMap (https://resistancemap.cddep.org/AntibioticUse.php) and World Health Organization websites (Supplementary Data 5). Other important information (e.g., sanitation and drinking water supply) was not completely available for our datasets, and could not be included in our analysis.

### Statistics and reproducibility

The gene prevalence in a specific biome was determined by dividing the number of samples in the biome where the targeted ARGs were detected by the number of total screened samples in the biome. To compare the prevalence of genes across different biomes, Fisher’s exact test (two.sided) was performed in the package “stats” in R (v.3.6.1). To evaluate how ARGs distribution differed across different biomes, collection locations, collection years or potential hosts, cluster analysis was conducted based on Bray–Curtis distance with the method = “average” and permutational multivariate analysis of variance was conducted with method = “Bray” and unconstrained permutation = 999, in the R package “vegan.” To evaluate the effects of biomes, collection locations and year, potential hosts and anthropogenic factors on ARGs distribution, redundancy analysis was conducted using the R package “vegan.” To evaluate whether the observed ARGs distribution (based on number of gene hits) across biomes was stochastic or deterministic, a null model approach was used to shuffle hits of individual ARGs across all screened biomes and generated 999 random distributions, by using the function randomizeMatrix (null.model = “frequency”, iterations = 1000) in the R package “picante.” The function mantel.test (nperm = 999, alternative = “two.sided”) in the R package “ape” was used to compare the observed and random distribution of ARGs. Bayesian analysis^43^ was used to track the potential transmission of ARGs across biomes, as previously used^4,44^. ARGs hits across samples and biome groups of these samples were used as input for Bayesian analysis with default parameters (e.g., rarefaction = 1000, train_rarefaction = 1000 and iteration = 100), based on a hypothesis that any biomes can be the source biomes that transmit ARGs to other biomes, as well as the sink biomes that accept ARGs from other biomes directly or indirectly. The Gini index evaluating the evenness of distribution was calculated in the R package “ineq.” The geographic distribution maps of genes with available collection locations were created using the R packages “ggplot2” and “scatterpie.” The longitude and latitude information of countries were utilized through the function map_data(‘world’) in the R package “ggplot2.” Heatmap was visualized by the R package “pheatmap” and other plots were visualized by the R package “ggplot2.”

### Reporting summary

Further information on research design is available in the Nature Portfolio Reporting Summary linked to this article.

## Supplementary information

Supplementary Information

Description of Additional Supplementary Files

Supplementary Data 1-10

Reporting Summary

## Data Availability

The public databases screened in this study are listed in Supplementary Data 3, and the metadata of the datasets are shown in the Supplementary Data 4. Source data underlying Figs. 1a, 2a, 3a and 4a, b are provided in Supplementary Data 6–10, respectively.
