# Supplementary material for: Screening of global microbiomes implies ecological boundaries impacting the distribution and dissemination of clinically relevant antimicrobial resistance genes
Source: Commun Biol. 2022 Nov 18;5:1217. doi: 10.1038/s42003-022-04187-x (PMC9674584; doi:10.1038/s42003-022-04187-x)
Supplement: Supplementary file 1 — Supplementary Information [file 42003_2022_4187_MOESM1_ESM.pdf]

Supplementary Information

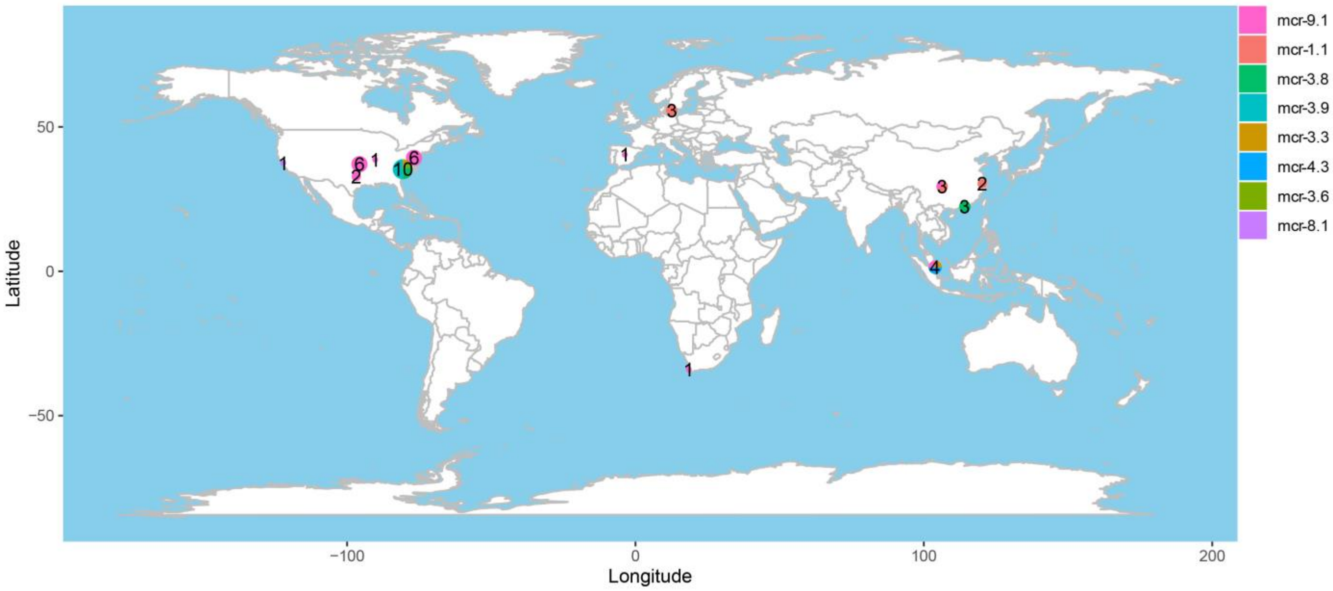

Supplementary Fig. 1 Global distribution of detected *mcr* genes. The number and size of pies provide hit number of *mcr* genes per location. The very nearby locations are combined and shown in the map. The rank of *mcr* genes in the legend is based on the decreasing hit number of individual genes. Only samples with available location information are included in the map.

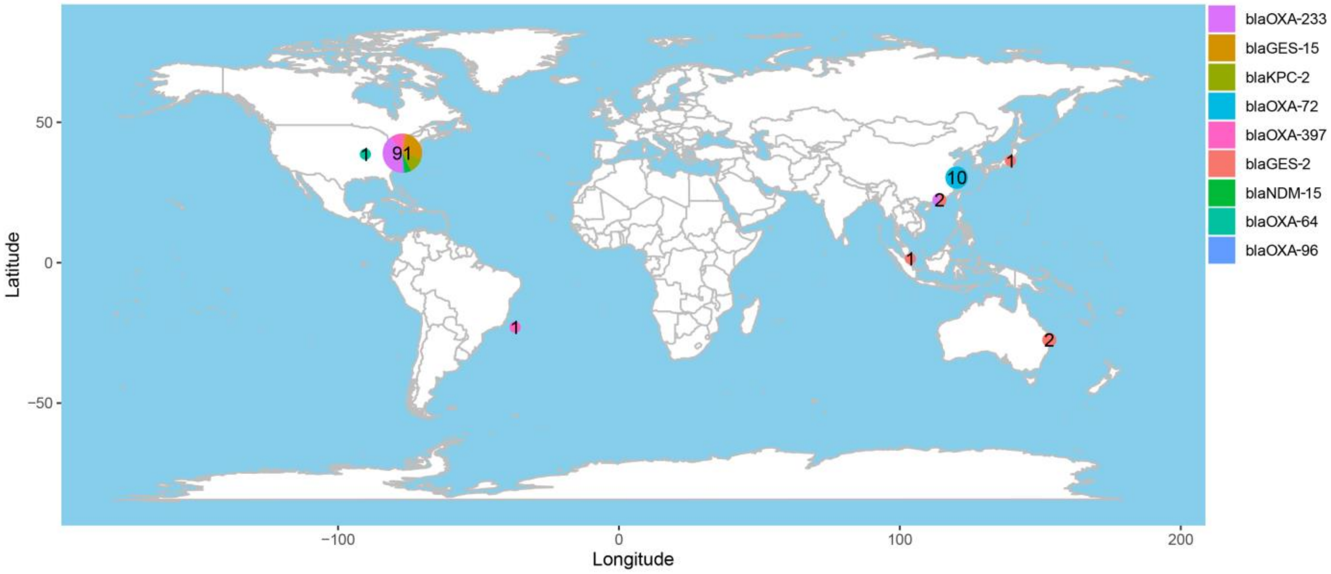

Supplementary Fig.2 Global distribution of detected CR genes. The number and size of pies provide hit number of CR genes per location. The very nearby locations are combined and shown in the map. The rank of CR genes in the legend is based on the decreasing hit number of individual genes. Only samples with available location information are included in the map.

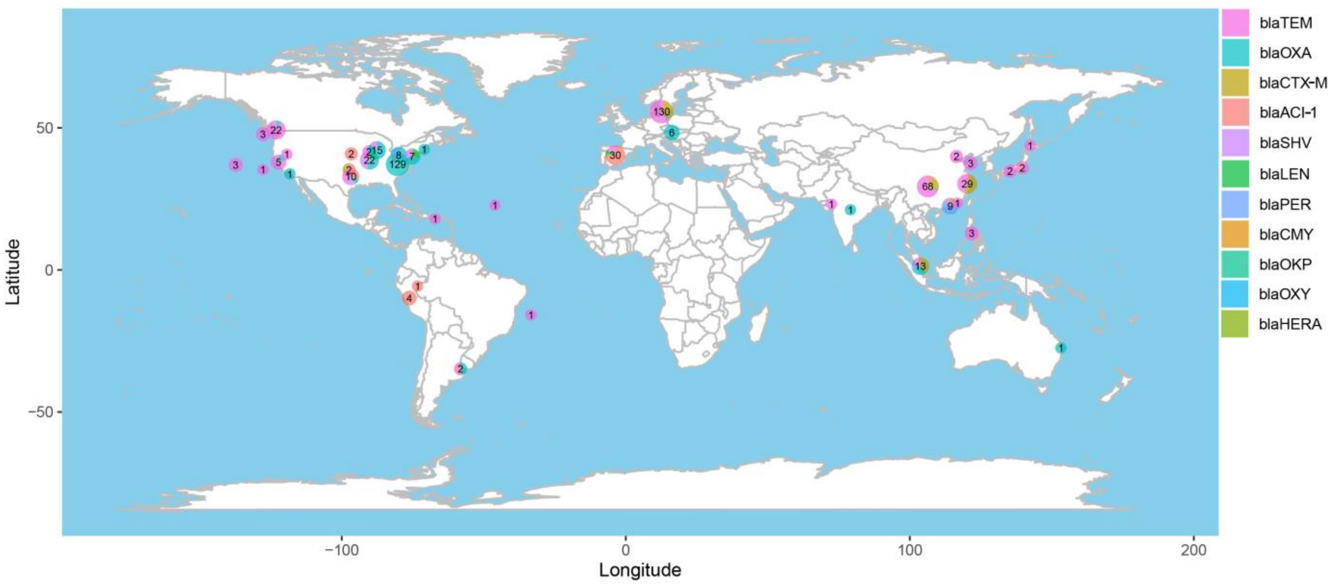

Supplementary Fig. 3 Global distribution of detected ESBL/BL (ESBL or BL) genes. The number and size of pies provide hit number of ESBL/BL genes per location. The very nearby locations are combined and shown in the map. The rank of ESBL/BL genes in the legend is based on the decreasing hit number of individual genes. Only samples with available location information are included in the map.

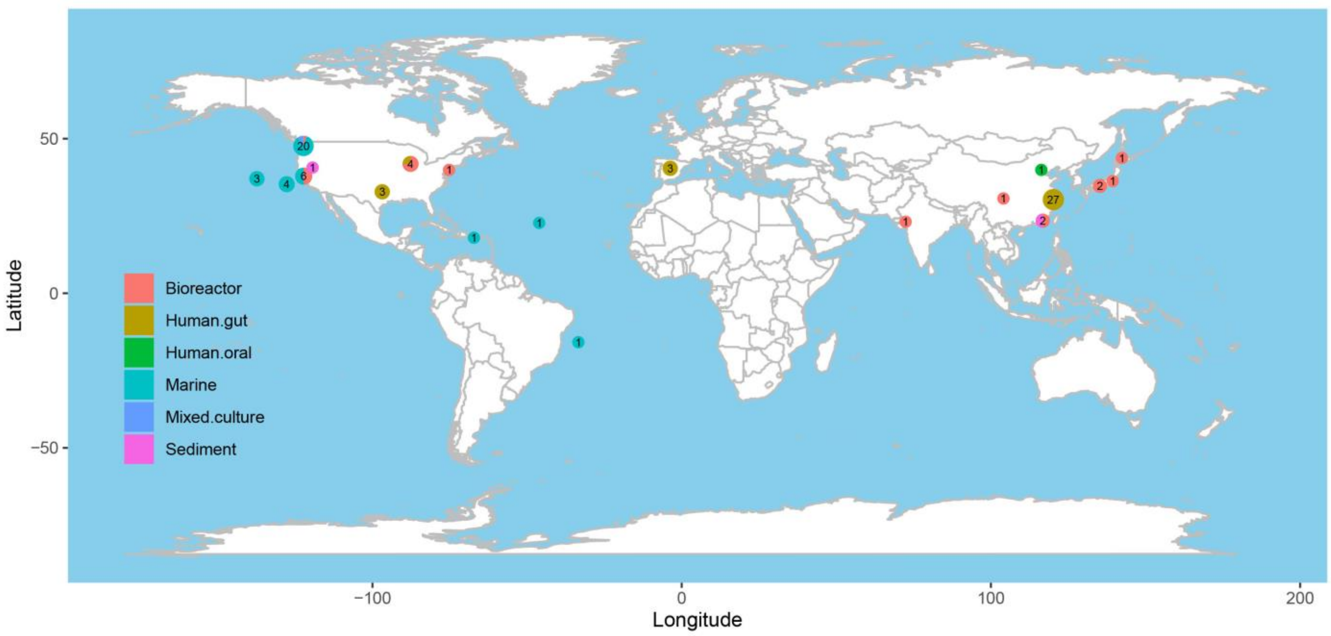

Supplementary Fig. 4 Global distribution of *blaTEM-116* genes. The number and size of pies provide hit number per location. The very nearby locations are combined and shown in the map.

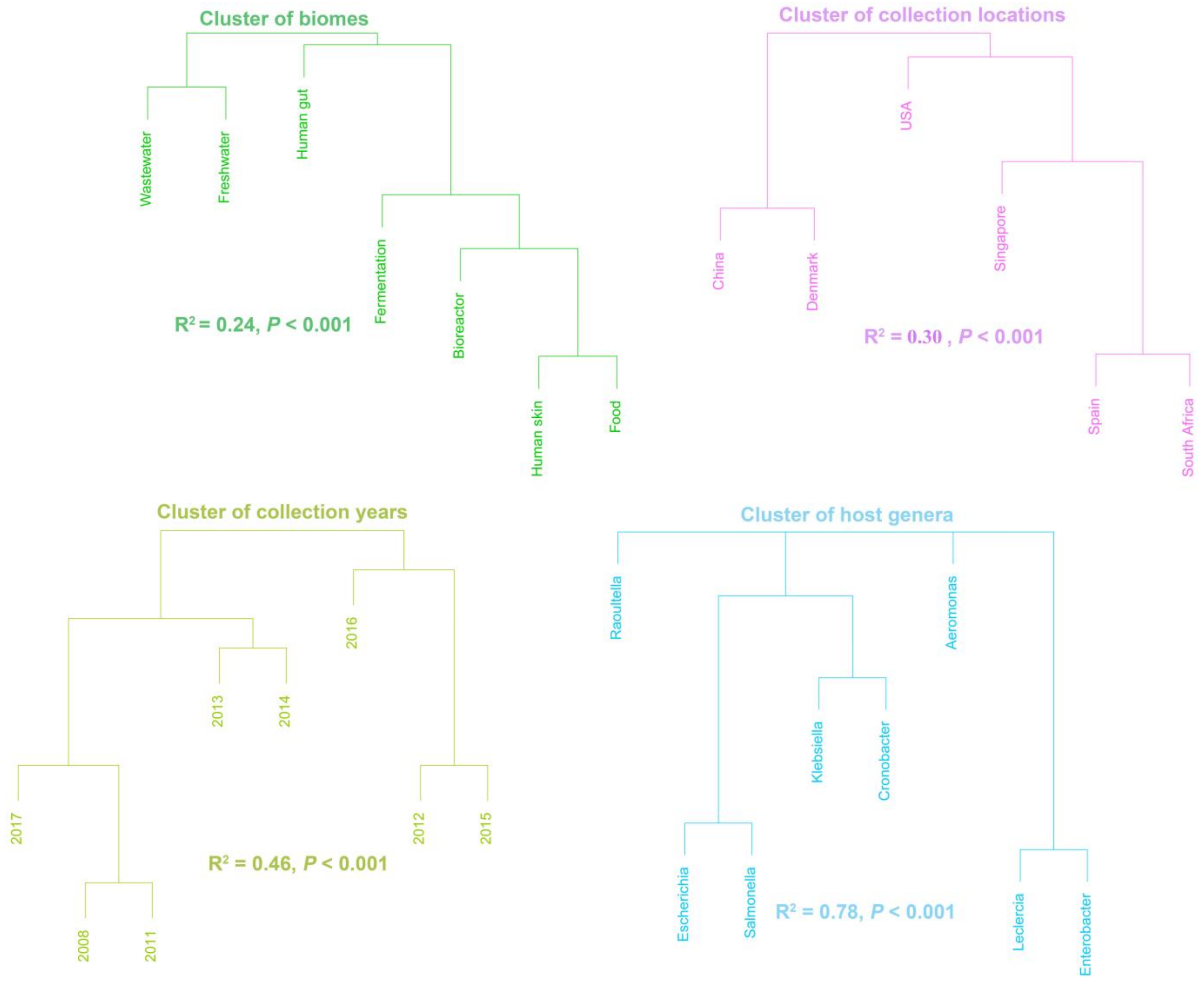

Supplementary Fig. 5 Dendrograms show the *mcr* genes clustering across biomes, collection locations, collection years and host genera, respectively. The values of R^2 and P in the permutational multivariate analysis of variance are shown below the dendrograms.

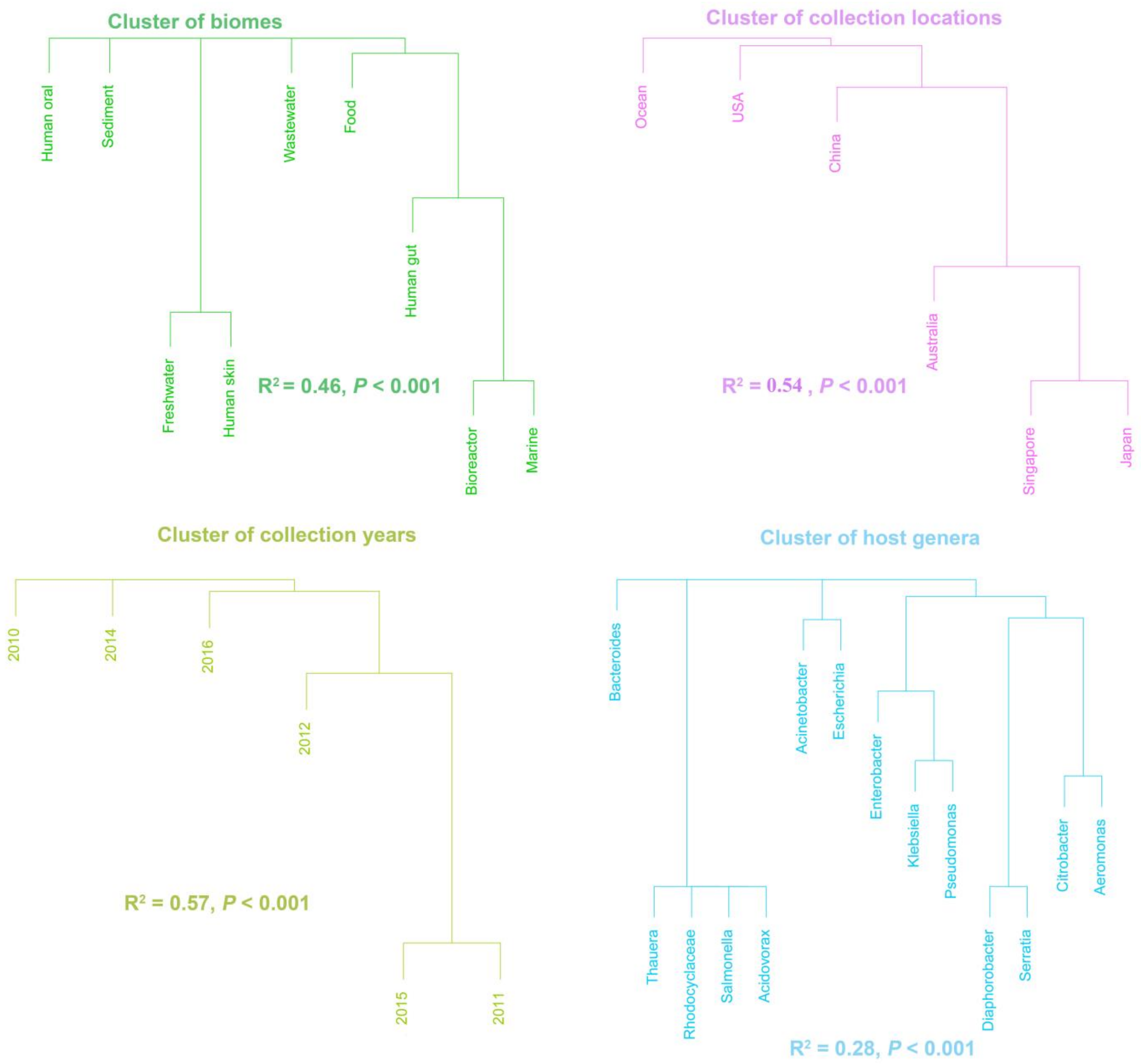

Supplementary Fig. 6 Dendrograms show the CR genes clustering across biomes, collection locations, collection years and host genera, respectively. The values of R^2 and *P* in the permutational multivariate analysis of variance are shown below the dendrograms.

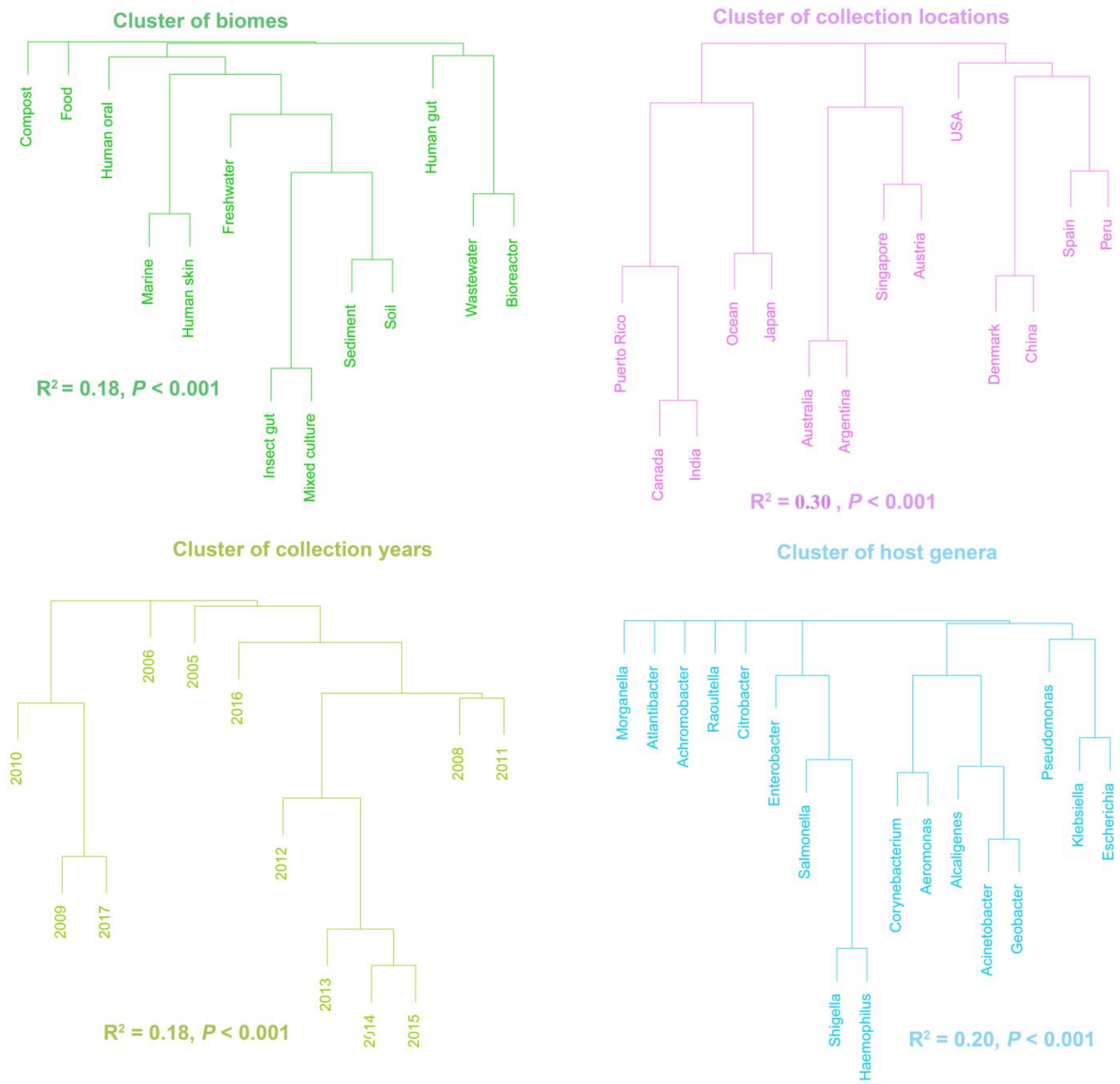

Supplementary Fig. 7 Dendrograms show the BL/ESBL genes clustering across biomes, collection locations, collection years and host genera, respectively. The values of R^2 and P in the permutational multivariate analysis of variance are shown below the dendrograms.

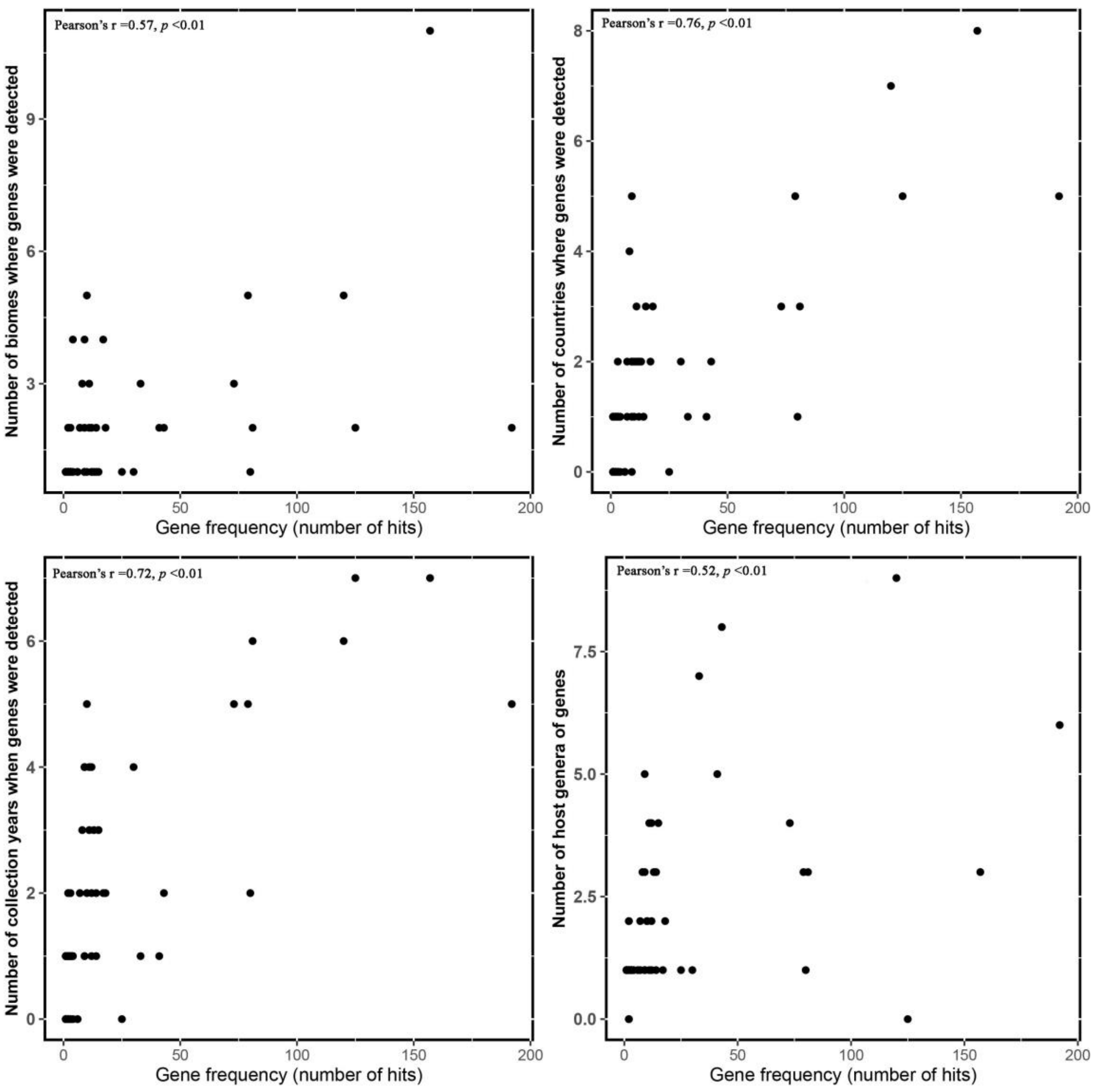

Supplementary Fig. 8 The relationship between gene frequency and numbers of biomes, collection years and host genera. Each point represents an individual gene variant from all detected resistance genes (*mcr*, CR, BL/ESBL). Spearman's correlation between the x-axis and y-axis in each sub-plot is conducted.

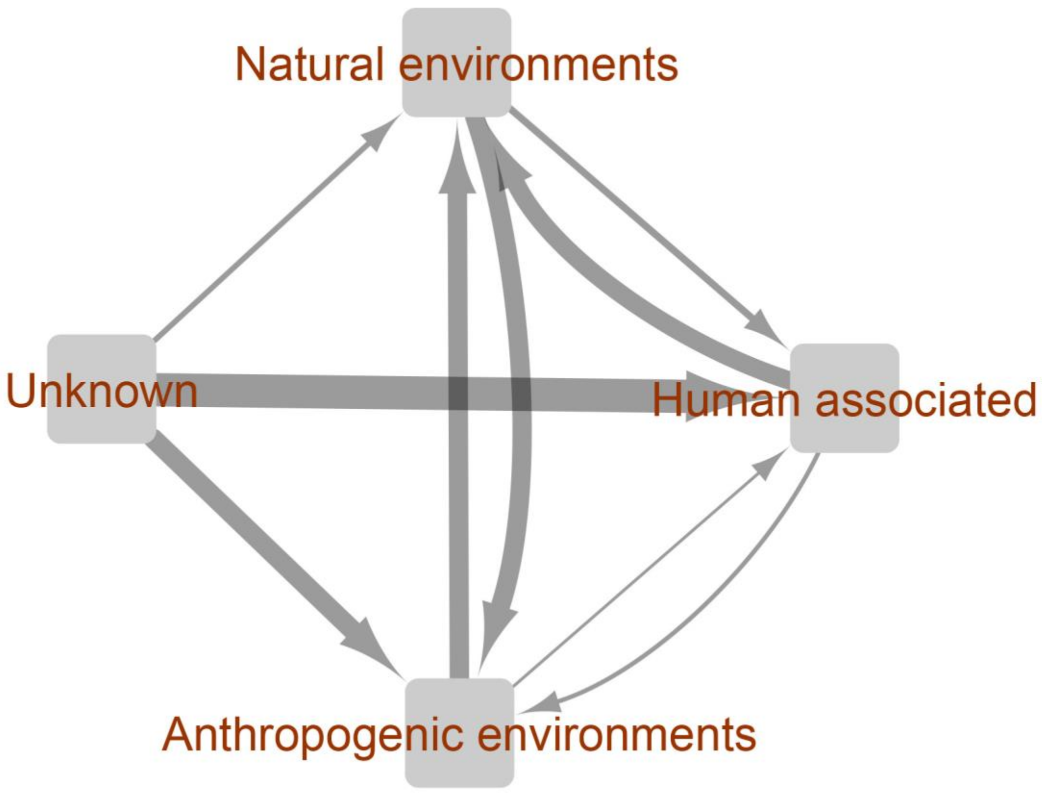

Supplementary Fig. 9 The Bayesian inference network portrays the dissemination of all the detected resistance genes across natural (freshwater, marine, sediment, insect gut and soil), anthropogenic (wastewater, bioreactor, compost, fermentation, food and mix-culture) and human-associated (human gut, oral and skin) environments. Based on a hypothesis that biomes can be the source that transmits resistance genes to other biomes, as well as the sink that accepts resistance genes from other biomes directly or indirectly, an arrow is used to represent the dissemination path from the source biomes to the sink biomes. Line width is based on the estimated transmission proportion of ARGs from the source biome to the sink biome, and the rectangle representing biome is sized by the number of resistance gene variants harbored in the biome. “Unknown” means no specific sources.

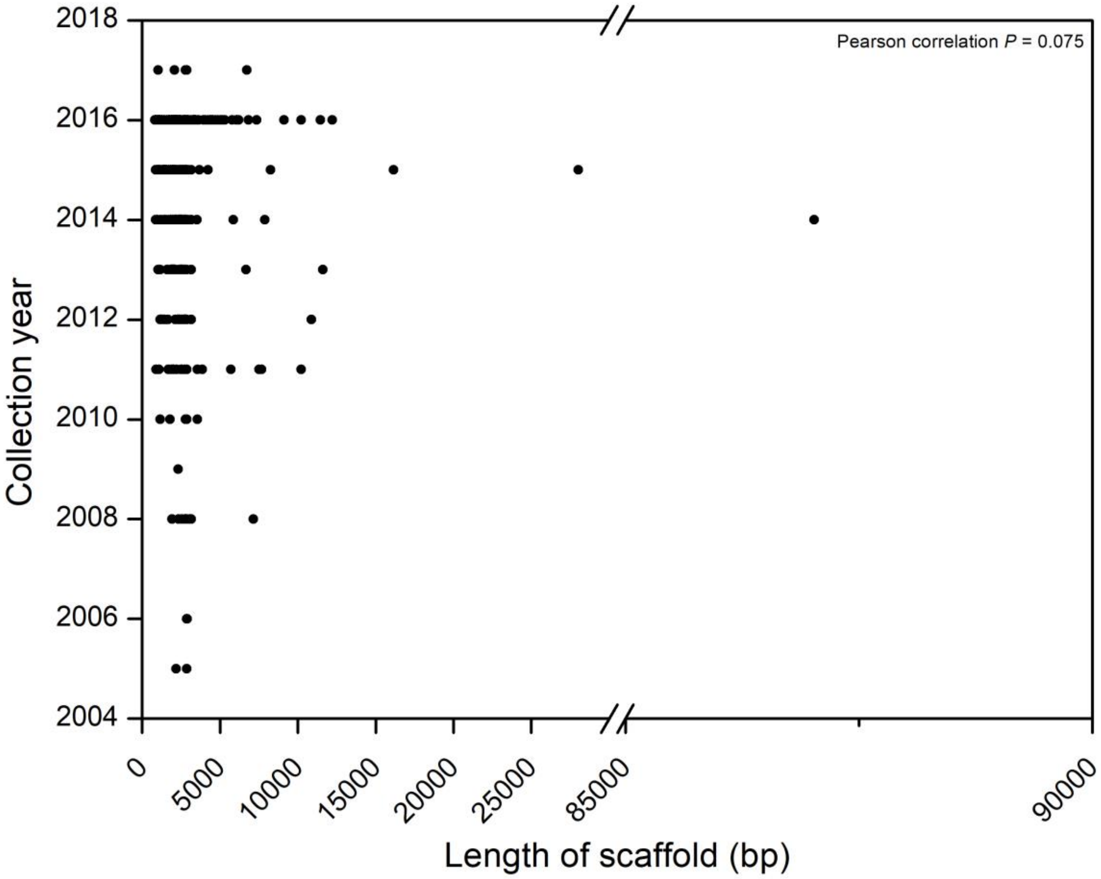

Supplementary Fig. 10 The relationship between length of scaffold and collection years samples that were detected to harbor *mc*r, CR or ESBL/BL genes.
