# Supplementary material for: Screening of global microbiomes implies ecological boundaries impacting the distribution and dissemination of clinically relevant antimicrobial resistance genes
Source: Commun Biol. 2022 Nov 18;5:1217. doi: 10.1038/s42003-022-04187-x (PMC9674584; doi:10.1038/s42003-022-04187-x)
Supplement: Supplementary file 2 — Description of Additional Supplementary Files [file 42003_2022_4187_MOESM2_ESM.pdf]

Description of Additional Supplementary Files

File name: Supplementary Data 1

Description: The effects of anthropogenic factors on the distribution of ARGs, evaluated by permutational multivariate analysis of variance with 999 permutations.

File name: Supplementary Data 2

Description: Queried resistance genes

File name: Supplementary Data 3

Description: Public databases and data sources screened in this study

File name: Supplementary Data 4

Description: Information of scaffolds

File name: Supplementary Data 5

Description: Anthropogenic factors of specific countries in specific years

File name: Supplementary Data 6

Description: Source data for Fig 1a

File name: Supplementary Data 7

Description: Source data for Fig 2a

File name: Supplementary Data 8

Description: Source data for Fig 3a

File name: Supplementary Data 9

Description: Source data for Fig 4a

File name: Supplementary Data 10

Description: Source data for Fig 4b
